# Evolution and Spatiotemporal Expression of *ankha* and *ankhb* in Zebrafish

**DOI:** 10.3390/jdb12030023

**Published:** 2024-09-09

**Authors:** Nuwanthika Wathuliyadde, Katherine E. Willmore, Gregory M. Kelly

**Affiliations:** 1 Department of Biology, Western University, London, ON N6A 5B7, Canada; nwathuli@uwo.ca; 2 Department of Anatomy and Cell Biology, Western University, London, ON N6A 5C1, Canada; katherine.willmore@schulich.uwo.ca

**Keywords:** *ANKH* genes, phylogeny, paralog, gene expression, bone mineralization, craniometaphyseal dysplasia, zebrafish

## Abstract

Craniometaphyseal Dysplasia (CMD) is a rare skeletal disorder that can result from mutations in the *ANKH* gene. This gene encodes progressive anksylosis (ANK), which is responsible for transporting inorganic pyrophosphate (PPi) and ATP from the intracellular to the extracellular environment, where PPi inhibits bone mineralization. When ANK is dysfunctional, as in patients with CMD, the passage of PPi to the extracellular environment is reduced, leading to excess mineralization, particularly in bones of the skull. Zebrafish may serve as a promising model to study the mechanistic basis of CMD. Here, we provide a detailed analysis of the zebrafish Ankh paralogs, Ankha and Ankhb, in terms of their phylogenic relationship with ANK in other vertebrates as well as their spatiotemporal expression patterns during zebrafish development. We found that a closer evolutionary relationship exists between the zebrafish Ankhb protein and its human and other vertebrate counterparts, and stronger promoter activity was predicted for *ankhb* compared to *ankha.* Furthermore, we noted distinct temporal expression patterns, with *ankha* more prominently expressed in early development stages, and both paralogs also being expressed at larval growth stages. Whole-mount in situ hybridization was used to compare the spatial expression patterns of each paralog during bone development, and both showed strong expression in the craniofacial region as well as the notochord and somites. Given the substantial overlap in spatiotemporal expression but only subtle patterning differences, the exact roles of these genes remain speculative. In silico analyses predicted that Ankha and Ankhb have the same function in transporting PPi across the membrane. Nevertheless, this study lays the groundwork for functional analyses of each *ankh* paralog and highlights the potential of using zebrafish to find possible targeted therapies for CMD.

## 1. Introduction

Craniometaphyseal Dysplasia (CMD) is a rare disorder characterized by excessive mineralization of the skull and long bones [1]. The clinical features of this disease include flared long bone metaphyses and several skull anomalies such as a wide nasal bridge, paranasal bossing, increased bizygomatic width and a prominent mandible [2]. Though less obvious phenotypically, the most severe functional consequences of CMD arise from the progressive thickening of the cranial base. This excess mineralization can occlude the foramina through which the cranial nerves pass and lead to facial palsy, hearing loss and blindness [3]. Unfortunately, there is no permanent cure for CMD, and the current treatment of the disease involves repetitive surgical recontouring of the affected bones [4]. Thus, further research is required to understand the mechanistic basis of CMD and to uncover potential therapeutic targets that can be used to prevent or permanently treat this disorder.

Genetic screens of individuals with CMD have determined that the disease can be caused by autosomal recessive mutations in the gene encoding the gap junctional protein connexin 43, but most commonly it is caused by autosomal dominant mutations in the gene progressive ankylosis homolog (*ANKH*) [5]. *ANKH* encodes a twelve-pass transmembrane protein (ANK) and is expressed in tissues throughout the body including skeletal tissues, consistent with the bony phenotypes present in patients with CMD [6]. The function of ANK is to transport intracellular ATP and inorganic pyrophosphate (PPi) into the extracellular environment [7]. Within the extracellular environment, ATP can be hydrolyzed into AMP and PPi by ectonucleotide pyrophosphatase 1, further increasing the level of extracellular PPi (ePPi) [8]. ePPi acts as a potent inhibitor of bone mineralization, with low concentrations leading to excessive deposition of hydroxyapatite crystals [9,10]. Thus, through its role in transporting ATP and PPi to the extracellular environment, ANK helps to regulate ePPi concentration and ultimately bone mineralization [4,8,9,10]. In CMD patients with *ANKH* mutations, the transport function of ANK is disrupted, causing excessive mineralization [1]. Several *ANKH* mutations have been reported in the CMD population, but most involve single amino acid substitutions, insertions or deletions and are in the highly conserved cytosolic domain [6]. The most common mutation among patients with CMD involves the deletion of phenylalanine (position 377 in the polypeptide) [4,6,11]. While patient screening has deepened our understanding of the genetic basis of CMD, animal models enable researchers to determine the cellular and molecular processes that underlie this disease and provide a means for exploring potential therapeutic targets.

Mouse models have greatly improved our understanding of the role of Ank in vivo. Loss-of-function mouse models of Ank (*Ank^ank^*^/*ank*^ and *Ank^null^*^/*null*^) demonstrate skeletal abnormalities including progressive arthritis and joint fusion that have been attributed to disrupted PPi transport and increased hydroxyapatite deposition within the joints [12,13]. The knock-out mouse model (*Ank^null^*^/*null*^) mimics several characteristics observed in human CMD patients, such as thickened skull bones, a narrowed foramen magnum and fused ossicles of the middle ear [13]. However, some CMD phenotypes are not present in these mice, including overgrown mandibles, obstructed nasal sinuses and flared long bone metaphyses [4,6,11]. Thus, Chen and colleagues developed a more targeted knock-in mouse model (*Ank^KI^*^/*KI*^) using an in-frame deletion of Phe377. Indeed, these mice closely phenocopy the symptoms observed in human CMD, including thick skull bones, a narrowed foramen magnum and fused middle ear bones, but additionally they demonstrate obliterated nasal sinuses, stenosis of cranial nerve foramina, widened long bone metaphyses and mandible hyperostosis [4]. While these mouse models have proven effective for helping to uncover the role of Ank and the pathogenesis of CMD, they are not perfect surrogates for the human disease. For example, in humans with CMD caused by mutations in *ANKH*, the disease arises in an autosomal dominant manner [6]. However, *Ank^KI^*^/*+*^ mice have skeletal phenotypes intermediate to those of wild-type (*Ank^+^*^/*+*^) and homozygous mutants (*Ank^KI^*^/*KI*^), with only *Ank^KI^*^/*KI*^ mutants demonstrating the phenotypic severity observed in patients suffering from CMD [4]. Additionally, joint stiffness observed in these mice has not been reported in CMD patients, indicating potential differences in disease manifestation between the mouse model and human CMD [4]. Moreover, mouse models are not as conducive to high-throughput therapeutic screens or studies of embryological pathogenesis of disease as other non-mammalian animal models. One such animal model commonly used to study bone diseases is the zebrafish [14].

Zebrafish offer convenience and relative cost-effectiveness compared to rodent models. For example, zebrafish produce a large clutch of embryos that are relatively transparent, develop rapidly and can be easily manipulated [15]. Additionally, the zebrafish genome is sequenced and annotated and can be manipulated using knock-out, knock-down and knock-in models of the genes of interest to generate targeted tissue dysfunction [14,15,16,17,18]. Zebrafish have also proven to be an effective model organism for bone disease studies [14,18,19], with bone mineralization beginning five days post-fertilization (dpf) [14]. Thus, the utility of the zebrafish is well known, and this model could be exploited as a useful tool for studying the pathogenesis of CMD and targeted drug screening for this disease.

However, zebrafish possess two paralog *ankh* genes, *ankha* and *ankhb*, that emerged during a duplication event [10,20]. Given this gene duplication, a better understanding of the origin and evolutionary relationship of the *ankha* and *ankhb* paralogs with *ANKH*/*Ank* of other vertebrates, as well as their spatiotemporal expression patterns, is crucial to determine the efficacy of using zebrafish to model CMD. To that end, we constructed phylogenetic trees using established amino acid sequences, conducted promoter prediction and functional prediction analyses comparing the two paralogs in zebrafish and performed a comprehensive analysis of the spatiotemporal expression of these paralogs during bone formation. Together, our findings constitute the necessary first steps in determining the utility of a zebrafish model for CMD and provide a framework for future studies.

## 2. Materials and Methods

### 2.1. Sequence Alignment and Phylogenetic Tree Construction of ANKH Proteins

Phylogenetic trees were constructed to explore the evolutionary conservation and diversification of ANKH across vertebrates, from jawless fish to mammals, by obtaining and comparing their amino acid sequences from Ensembl [21]. The following sequences were used: human (*Homo sapiens*—ENSP00000284268.6), mouse (*Mus musculus*—ENSMUSP00000022875.7), rat (*Rattus norvegicus*—ENSRNOP00055036996.1), guinea pig (*Cavia porcellus*—ENSCPOP00000011102.2), rhesus macaque (*Macaca mulatta*—ENSMMUP00000080342.1), pig (*Sus scrofa*—ENSSSCP00000017779.3), domestic chicken (*Gallus gallus*—ENSGALT00010001547.1), tropical clawed frog (*Xenopus tropicalis*—ENSXETP00000100546.1), zebrafish (*Danio rerio*—ENSDARP00000105177.1 and ENSDARP00000002526.7), common carp (*Cyprinus carpio*—ENSCCRT00000085173.2 and ENSCCRT00000029117.2), Mexican tetra (*Astyanax mexicanus* ENSAMXG00000002808 and ENSAMXT00000038996.1), inshore hagfish (*Eptatretus burgeri*—ENSEBUG00000011035), Sea lamprey (*Petromyzon marinus*—ENSPMAT00000002489.1) and Elephant shark (*Australian ghostshark*) (*Callorhinchus milii*—ENSCMIT00000028310.1). The phylogenetic trees were constructed using neighbor-joining analyses to assess the taxonomic relationships of ANKH. The trees were rooted with inshore hagfish (*primitive species*) and were based on a multiple sequence alignment using MEGA version 10. The analysis was conducted with 10,000 bootstrap replicates [22].

### 2.2. Percentage Identity Matrix

The percentage identity matrix quantifies and visually presents the sequence identity between the ANKH amino acid sequences across different vertebrate species. This matrix was created by analyzing the amino acid sequences specified in Section 2.1. The amino acid sequences were aligned and then processed using software (EMBL-EBI, Hinxton, UK) [23], which calculated the percentage identity matrix. These percentage identity values were organized in a matrix format, facilitating the visualization of the relationships between the ANKH amino acid sequences across various species.

### 2.3. Promoter Prediction Analysis

Promoter prediction analysis was conducted to identify the gene regulatory regions and to understand the expression control of the *ANKH*, *Ank* and *ankh* genes in human, mouse and zebrafish, respectively. The sequences were downloaded from Ensembl, and the 5′ flanking regions were used as input files for determining the transcriptional start site (TSS) of each gene. The Neural Network Promoter Prediction (NNPP version 2.2) toolset was used with the minimum standard predictive score (ranging between 0 and 1) available at https://www.fruitfly.org/seq_tools/promoter.html, (accessed on 9 May 2024). The highest prediction score was considered for regions containing more than one TSS.

### 2.4. Gene Ontology (GO) Annotation Analysis

The Gene Ontology (GO) annotation analysis was conducted to predict the biological significance of the ANKH proteins. The amino acid sequence of ANKH/Ank and Ankh for humans, mice and zebrafish was downloaded from Ensembl, and functional prediction analysis was conducted using InterPro software (Interpro Translation Solutions, Wheaton, IL, USA) [24].

### 2.5. Zebrafish Maintenance

Mature Tübingen (TU) zebrafish were raised in the Department of Physiology and Pharmacology, Western University, London, ON. The fish were kept in an aquarium at a temperature of 28 °C, with a lighting schedule of 14 h of light and 10 h of darkness. The aquarium was supplied with fresh water and aeration to maintain its environment. The pH and conductivity were monitored daily and maintained at 7–8 and 200–3000 micro-Siemens, respectively. Alkalinity, hardness, carbon dioxide and dissolved oxygen were monitored weekly and maintained at 50–75 ppm, 100–200 ppm, 0–15 ppm and 6–8 ppm, respectively. The present study adheres to the ethical guidelines established by the Canadian Council on Animal Care and was approved by the Animal Care Committee at Western University.

### 2.6. mRNA Isolation, cDNA Synthesis and Reverse-Transcription PCR

To determine the temporal expression pattern of *ankha* and *ankhb*, we performed RT-PCR targeting 19 different developmental time points. Total mRNA was extracted from zebrafish, including samples at embryonic and larval developmental stages (2-cell (0.75 h post-fertilization (hpf), 256-cell (2.5 hpf), sphere (4.0 hpf), dome (4.3 hpf), 50% epiboly (5.3 hpf), shield (6.0 hpf), 75% epiboly (8.0 hpf), 5–9-somite (11.0 hpf), 14–19-somite (16.0 hpf), 26+-somite (22.0 hpf), prim-5 (25.0 hpf), prim-15 (32.0 hpf), prim-25 (35 hpf), 2-dpf, 3-dpf, 4-dpf, 5-dpf, 6-dpf and larval day 7 stages). A pool of 30 embryos or larvae was collected for each stage, and mRNA extraction was performed using TRIzol reagent and chloroform (Thermo Fisher Scientific, Mississauga, ON, Canada). The isolated RNA was reverse-transcribed into cDNA using the High-Capacity cDNA Reverse Transcription kit (Thermo Fisher Scientific, Mississauga, ON, Canada), following the manufacturer’s instructions. RT-PCR was performed under the following reaction conditions: 500 nanomolar (nM) forward and reverse primers, 25 μL of DreamTaq Green PCR Master Mix (Thermo Fisher Scientific, Mississauga, ON, Canada), and 1 μL of cDNA template. The reactions were performed using a C1000 Touch Thermal Cycler (Bio-rad, Hercules, CA, USA) for 35 cycles. The samples were visualized using a 2% agarose gel (1× TAE) containing RedSafe, and the gels were imaged using the ChemiDoc Touch Imaging System. Primer sets for *ankha* and *ankhb* were utilized in the RT-PCR analysis (Table 1). *lsm12b* served as a loading control due to its consistent expression pattern compared to that of most other housekeeping genes [25]. All primers were optimized, and the desired amplicon was sent for sequencing for further confirmation.

### 2.7. Quantitative PCR (RT-qPCR)

RT-qPCR was conducted to quantify the gene expression patterns of *ankha* and *ankhb* in zebrafish samples at 2 dpf, 3 dpf, 4 dpf and 7 dpf, as these developmental stages represent times preceding and during bone formation. cDNA preparation was conducted as described above, and the primers and PCR conditions were selected in accordance with those previously described (Table 2) [26]. The comparative Ct method, indicated as 2^(−ΔΔCt)^, was used to analyze the relative gene expression and provided a ratio relative to the expression of constitutively active *gapdh*. We chose *gapdh* as our reference gene for this assay because a preliminary analysis indicated that during this specific developmental window, *gapdh* is more consistently expressed than *lsm12b* [20].

### 2.8. RNA Probes for In Situ Hybridization

Primers for *ankha* and *ankhb* were designed for in situ hybridization, and restriction enzyme sites were included for directional cloning (Table 3). Riboprobes were synthesized from amplified *ankha* and *ankhb* fragments, digested using EcoRI and HindIII and then ligated into the restriction enzyme-digested Bluescript SK+ plasmid. Following transformation into *E. coli* (DH5α), colonies were selected on LB/agar plates containing ampicillin and subsequently identified using colony PCR. Finally, restriction enzyme digestions were performed after the plasmids were isolated using a midiprep kit. All riboprobes were generated from linearized plasmids and synthesized using a DIG RNA Labelling kit (Sigma-Aldrich, Oakville, ON, Canada).

### 2.9. Whole-Mount In Situ Hybridization (WISH)

In situ hybridization was performed to study the spatial expression patterns of *ankha* and *ankhb* genes in zebrafish larvae at 2 dpf, 3 dpf and 4 dpf, fixed in 4% paraformaldehyde using an established protocol [27]. Briefly, the fixed samples were incubated with a hybridization solution containing sense or antisense riboprobes. This step was followed by thorough washing, blocking with 5% heat-inactivated sheep serum and incubation with an anti-digoxigenin antibody conjugated to alkaline phosphatase. NBT/BCIP (4-nitro blue tetrazolium chloride/5-bromo-4-chloro-3-indolylphosphate) was added after thoroughly washing the samples, and staining was carried out in the dark until a signal was detected in the embryos/larvae incubated with the sense-strand probes. An antisense *krox-20* riboprobe was used for zebrafish at 24 hpf as a positive control. All samples were cleared in glycerol and stored in PBS–Tween 20. Images were collected using a Nikon Inverted T12F Deconvolution Microscope (Nikon, Mississauga, ON, Canada).

## 3. Results

### 3.1. Zebrafish ankhb Demonstrates a Closer Evolutionary Relationship to ANKH Proteins of Other Vertebrates

**Figure 1:**
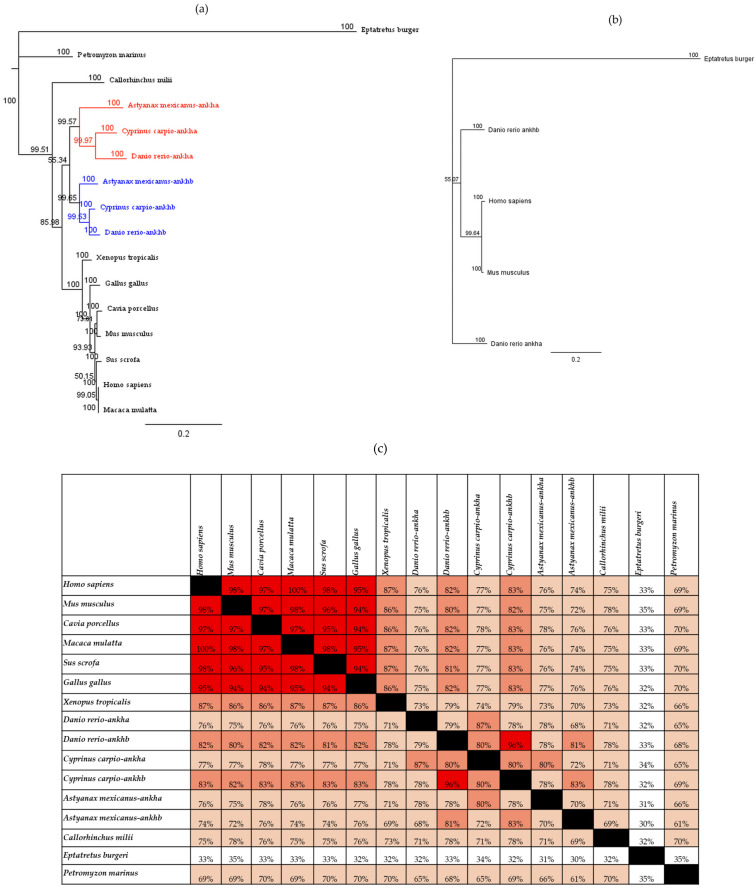
Phylogenetic relationships of the ANKH amino acid sequence in vertebrate organisms. (**a**) Results from neighbor-joining tree analysis using the out-group taxon (*Eptatretus burgeri*) demonstrate the relationships between vertebrate organisms. Bootstrap analysis was performed using 10,000 replicates, and these bootstrap values are included. Monophyletic clustering of teleost fish for Ankha and Ankhb are represented by red and blue branches, respectively. (**b**) The neighbor-joining tree analysis includes human (*Homo sapiens*), mouse (*Mus musculus*), and zebrafish (*Danio rerio*) Ankha and Ankhb, and the out-group taxon (*Eptatretus burgeri*) includes values from 10,000-replicate bootstrap analysis. (**c**) Matrix of pairwise amino acid sequence identities for Ankh/Ankha and Ankhb across various vertebrate species determined using percentage identity matrix analysis. Boxes with a black background that have no values represent self-comparisons. High sequence identity is represented by red, and white shades highlight comparisons with lower sequence identity. Squares that have 90 to 100% identity are shown with a dark pink background, sequence identity values from 80–90% are represented in light pink, sequence identity values from 50–80% in white, and sequence identity values below 50% are represented with a white background. Each identity value is rounded to the nearest whole number.

Our first goal was to identify which of the zebrafish ankh proteins was more closely related to the single protein present in other vertebrates including rodents and humans. The resulting neighbor-joining tree topology was aligned (Figure 1a), and the tree was rooted with the jawless fish species inshore hagfish (*Eptatretus burgeri*). Within this tree, the elephant shark/Australian ghostshark (*Callorhinchus milii*), representing cartilaginous fish, branched off separately from the other vertebrate group. Teleost species including zebrafish (*Danio rerio*), Mexican tetra (*Astyanax mexicanus*), and the common carp (*Cyprinus carpio*) formed a cohesive cluster with distinct monophyletic groups for Ankha and Ankhb. All other vertebrates appeared positioned on the phylogenetic tree in accordance with the established vertebrate phylogeny [28].

To further elucidate the relationship between zebrafish Ankha and Ankhb and their counterparts in humans and mice, a neighbor-joining phylogenetic tree was constructed using these four sequences (Figure 1b). Zebrafish Ankhb clustered within the same monophyletic group as the sequences from humans and mice, underscoring their close evolutionary relationship. In contrast, zebrafish Ankha appeared more distantly related to rodents and humans. The percent identity values confirmed this observation (Figure 1c), revealing 82% amino acid sequence identity between zebrafish Ankhb and human ANK and 80% identity between zebrafish Ankhb and the mouse protein. In comparison, Ankha had 76% and 75% sequence identity to that of human ANK and mouse Ank, respectively. These findings indicate that the Ankhb paralog in zebrafish is evolutionarily closer to the ANK/Ank in humans and mice. To determine which paralog originated earlier, we also compared the amino acid sequences of Ankha and Ankhb with the amino acid sequence of Ankh from species that evolved before zebrafish. We did not identify Ankh in any invertebrate species on the Ensembl site, but the Ankh protein was identified in jawless fish species, specifically, *Petromyzon marinus* and *Eptatretus burgeri*. Additionally, *Callorhinchus milii*, a cartilaginous fish, also harbors a single ankh protein, but evidence indicates that it would be characterized as Ankhb. *Eptatretus burgeri* exhibits the same level of identity (≈32%) for both Ankha and Ankhb, whereas *Petromyzon marinus* possessed only one *Ankh* protein. Furthermore, *Petromyzon marinus* Ankh demonstrated a higher amino acid sequence identity to zebrafish Ankhb (Figure 1c). In summary, zebrafish Ankhb demonstrated a closer evolutionary relationship to the ANK proteins of rodents and human and appears to represent the more primitive lineage among the Ankh family.

### 3.2. Differential Promoter Strength in Zebrafish ankh Paralog Genes

The transcriptional start sites (TSSs) were predicted for four *ANKH* genes (human, mouse, and zebrafish). For genes with two or more TSSs, the one with the highest predictive score was considered the optimal TSS location. All predicted TSSs for these genes had a score ranging from 0.80 to 1.00 and are presented in Table 4. The predictions indicated that human *ANKH* has TSSs ranging from 1 to 5, with the highest promoter prediction score of 0.87, located between 461 and 511 bp. For mouse, two TSSs were detected, each with a predictive score of 0.83 (in the 430–480 bp range and 515–565 bp range). The *ankha* gene in zebrafish, which has four TSSs, showed the highest promoter prediction score of 0.91 (115–165 bp), whereas *ankhb* had a single detected TSS with a predictive score of 0.96, ranging from 215 to 265 bp. The fact that *ankhb* had the highest predictive score for its single TSS is indicative of its most robust promoter activity among the genes studied and suggests a more consistent and strong transcriptional regulation compared to *ankha*.

### 3.3. Similar Functional Biology of ANKH Proteins in Humans, Mice and Zebrafish

GO annotations were conducted for human, mouse, and zebrafish Ankha and Ankhb proteins to identify and compare their biological significance (Table 5). The biological process (GO:0035435) predicted the same function for all four proteins of phosphate ion transmembrane transport. The molecular function (GO:0005315) of the four proteins suggested that all have inorganic phosphate transmembrane transporter activity. Furthermore, the predicted cellular component (GO:0016020) was identified as membrane-localized. Thus, these results suggest that all four proteins—ANKH, Ank, Ankha, and Ankhb—share the same biological process, molecular function and cellular component ontology.

### 3.4. ankha and ankhb Expression Patterns in Zebrafish Embryonic and Larval Development

**Figure 2:**
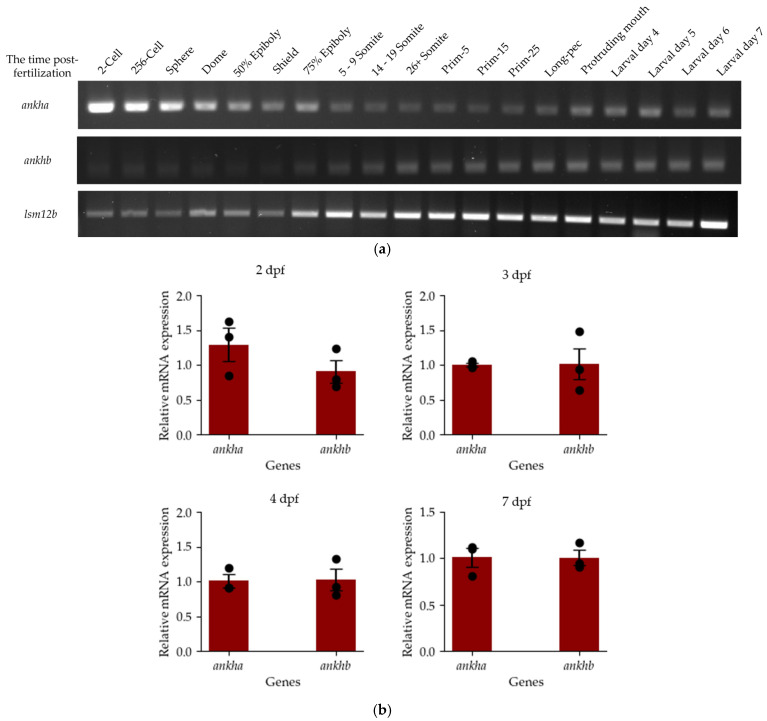
Temporal expression of *ankha* and *ankhb* during zebrafish development. (**a**) Expression of *ankha* and *ankhb* relative to the *lsm12b* control in this representative-endpoint PCR gel, *N* = 3 replicates. Thirty embryos or fry from each developmental stage, ranging from the 2-cell stage (0.75 hpf) to the 7 dpf stage, were sampled. (**b**) RT-qPCR normalized expression of *ankha* and *ankhb* genes at 2, 3, 4 and 7 days post-fertilization (dpf) relative to *gapdh* expression, showing the mean +/− the standard error. Each sample represents 30 pooled larvae, where *N* = 3, and statistical significance was determined using an unpaired *t*-test (*p* < 0.05).

**Figure 3:**
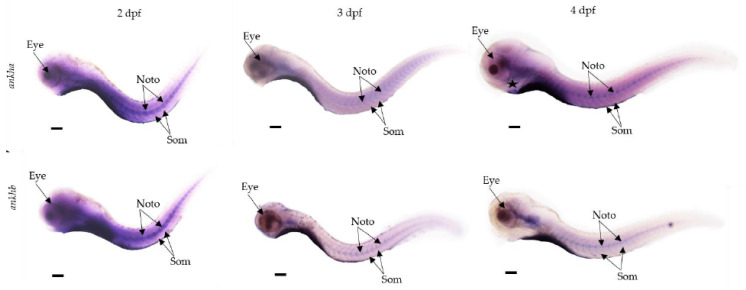
Whole-mount in situ hybridization analysis of *ankha* and *ankhb* spatial expression patterns in zebrafish development. Lateral views of zebrafish larval stages at 2, 3 and 4 days post-fertilization (dpf) reveal spatial gene expression patterns for *ankha* and *ankhb*. At 2 and 3 dpf, both *ankha* and *ankhb* show predominant staining in the craniofacial region, somites and notochord. At 4 dpf, *ankha* extends below the eye (star) and appears in the somites and notochord (arrows). *ankhb* expression at 3 dpf and 4 dpf is mainly localized to the craniofacial region and notochord area. For *ankhb*, staining below the eye is reduced and is more localized in regions below the hindbrain. Noto: notochord, Som: somites. The yolk was removed from the embryo; the scale bar indicates 100 μm.

RT-PCR was performed to investigate *ankha* and *ankhb* transcript expression in 19 different developmental stages, with *lsm12b* as a loading control (Figure 2a). The expression of each paralog was seen in all embryonic and larval stages of development, and Sanger sequencing confirmed that they represented *ankha* and *ankhb*. Amplicons for both genes were detected at the two-cell stage (0.75 hpf), showing that they are maternally derived and present before the midblastula transition [29]. Although little can be determined quantitatively based on amplicon intensity using this approach, the apparent intensity of the *ankha* amplicon was higher than that of *ankhb* at the two-cell stage and decreased after 75% epiboly (8 hpf). The intensity of *ankha* resumed in the larval developmental stages (2 dpf, 3 dpf, 4 dpf, and 7 dpf). Likewise, *ankhb* intensity gradually increased from the two-cell stage to the larval stages, but it should be noted that there was a decrease in the intensity of *ankha* and a concomitant increase in amplicon intensity for *ankhb* expression at 75% epiboly. Despite this noticeable difference, amplicons corresponding to both *ankha* and *ankhb* genes were detected at all stages of development. Since both genes are implicated in transporting Ppi across the membrane, a process necessary for bone mineralization, a quantified approach was used to detect gene expression during a temporal window corresponding with bone formation. Specifically, RT-Qpcr was used to assess *ankha* and *ankhb* expression at 2 dpf, a time just preceding bone formation, as well as in several stages during bone formation (3 dpf, 4 dpf, and 7 dpf) (Figure 2b). However, no significant differential expression between these paralogs was detected at these stages. Given that *ankha* and *ankhb* were expressed throughout embryonic and larval development, including stages corresponding to bone formation, and showed similar expression levels, we are unable to assign distinct developmental functions to each paralog. A limitation of our study is the potential masking of the differential expression between the *ankha* and *ankhb* genes seen in the RT-Qpcr results. Thus, in subsequent studies, it would be interesting to dissect cranial regions and probe them for differential changes as those evident from the in situ hybridization results shown in Figure 3.

### 3.5. Expression of *ankha* and *ankhb* Is Localized to the Craniofacial Region, Somites, Notochord and Tail

The spatial expression patterns of the *ankha* and *ankhb* genes during zebrafish larval development at 2 dpf, 3 dpf and 4 dpf were examined using DIG-labeled antisense probes and WISH (Figure 3). At 2 dpf, staining of *ankha* and *ankhb* was apparent near the developing eye and notochord, with non-specific staining throughout the body. By day 3, both *ankha* and *ankhb* expression remained in the craniofacial region, specifically, the brain, with somites and the notochord showing a more localized staining compared to the remainder of the body. At 4 dpf, the expression pattern of *ankha* extended rostrally below the eyes (star) and brain, with staining seen in the notochord and somites. The expression of *ankhb* at 4 dpf differed somewhat from that of *ankha*, with less intense staining observed in the brain and spinal cord, and no signal below the eyes. At 4dpf, staining for *ankhb* was also present in the notochord and somites, as seen for *ankha*.

### 3.6. Alignment of ANKH Proteins to Determine Their Homology to Known Mutations That Cause CMD in Humans

We conducted multiple sequence alignments of the ANK, Ank, Ankha and Ankhb proteins to determine their homology with known mutations that cause CMD in humans. The alignment showed a high degree of conservation among a wide variety of species. Interestingly, we observed that the six main mutations that cause CMD in the human population [6] are conserved in the zebrafish Ankha and Ankhb amino acid sequences (Supplementary Figure S1). This finding further validates the use of zebrafish as a model organism for studying CMD.

## 4. Discussion

Human CMD is a rare bone disorder caused by mutations in the *ANKH* gene [1]. There is no permanent cure for this disorder, and management of its symptoms is typically achieved through repeated invasive surgeries to recontours the affected bones [4,30]. Since zebrafish is an excellent system for studying bone diseases and can be utilized for high-throughput drug screening, a zebrafish model for CMD could provide much needed insight into the effectiveness of non-surgical treatment options for this debilitating disorder [14,15,16,19,31]. However, zebrafish have paralog genes for *ANKH* that are not well understood and have not been studied in detail. Therefore, prior to creating a zebrafish CMD model, it was necessary to examine the evolutionary patterns, conservation and spatiotemporal expression of the *ankha* and *ankhb* paralogs in zebrafish. Our study highlights two key findings: first, the Ankhb amino acid sequence in zebrafish shows high similarity to the ANK sequence in humans, underscoring the evolutionary conservation of this paralog; second, *ankha* and *ankhb* show similar spatiotemporal expression patterns, indicating their potential for functional redundancy and critical roles across various developmental stages.

### 4.1. The ankhb Amino Acid Sequence in Zebrafish Shows Greater Similarity to ANK Sequence in Humans

The phylogenetic trees we constructed shows the evolutionary history of the *ANKH* gene in vertebrates. Two whole-genome duplications during vertebrate evolution and a third event specific to teleost fish led to the presence of *ankha* and *ankhb* paralog genes in zebrafish [32,33]. The monophyletic clustering of Ankha and Ankhb with their respective paralogs in teleost species, such as common carp and Mexican tetra, aligns with the evolutionary pattern observed in other gene families, such as *dlx* [34]. These patterns indicate that gene duplication events in teleosts preserved distinct gene functions within paralog groups. Our findings that Ankhb clustered more closely with human and mouse ANKH proteins, supported by high amino acid sequence identity (82% with human ANK and 80% with mouse Ank), underscore the evolutionary conservation of Ankhb. This conservation suggests that Ankhb may retain its role in bone mineralization processes shared with human ANKH, reinforcing its relevance for modeling CMD. The presence of a single Ankh protein in species predating teleosts, such as jawless and cartilaginous fish, which also aligned more closely with Ankhb, further emphasizes the ancestral significance of this paralog.

Further supporting the relevance of Ankhb for human disease modeling, our promoter prediction analysis revealed significant differences in the transcriptional regulation of the *ankha* and *ankhb* genes in zebrafish. The *ankhb* gene, with a singular TSS and a high predictive score of 0.96, indicated strong and consistent promoter activity, likely resulting in stable and efficient gene expression, which is crucial for its role in bone mineralization processes [35]. In contrast, the *ankha* gene, with multiple TSSs and a lower predictive score of 0.91 compared to *ankhb*, indicated a more complex and variable promoter activity [36,37]. This variability might provide regulatory flexibility, allowing *ankha* to respond to diverse cellular conditions or developmental stages, but could also result in less consistent gene expression.

### 4.2. Both ankha and ankhb Exhibit Similar Spatiotemporal Expression Patterns, Suggesting Functional Redundancy

Zebrafish *ankha* and *ankhb* genes appeared actively expressed at all key developmental stages. At days 2–7, the expression aligned with bone mineralization in the craniofacial region and other areas such as vertebrae, operculum, and ceratobranchial bones [38]. This suggests that both *ankha* and *ankhb* might be involved in bone development, in agreement with the involvement of ANK/Ank protein in the transport of ATP and inorganic pyrophosphate (PPi) in humans and mice [7,8,39]. Earlier on, *ankha* and *ankhb* demonstrated maternal inheritance, with expression detected at the two-cell stage, prior to the midblastula transition. This early expression would indicate that these genes are maternally deposited and play crucial roles in the egg-to-embryo transition, potentially involving ATP transportation [40]. The period from 3 h post-fertilization (hpf) to 24 hpf in zebrafish is associated with a substantial rise in the levels of metabolites, including amino acids, nucleic acids, and energy metabolites like complex sugars and TCA cycle intermediates [41]. These intermediates likely contribute to increased levels of ATP and other metabolites, with *ankha* and *ankhb* transcripts potentially involved in their transport. Considering the high expression of human ANK in oocytes and its significant role in tissues requiring ATP [42], it is likely that zebrafish *ankha* and *ankhb* contribute to early energy transport. These findings imply that the effective modeling of CMD in zebrafish may require temporal-specific gene knock-out and knock-in mutants [43], with attention to off-target effects.

The spatial expression patterns of the *ankha* and *ankhb* genes during zebrafish larval development revealed their involvement in critical developmental regions. Both genes appeared prominently expressed at 2 dpf in the craniofacial region, somites, notochord and tail, with notable expression near the developing eye and notochord, indicating their role in early tissue formation. By day 3, *ankha* and *ankhb* expression became more localized, showing strong staining in the brain, somites and notochord. At 4 dpf, *ankha* expression extended in the brain and below the eye, particularly around the parasphenoid bone where osteoblasts would be differentiating, while *ankhb* showed less intense staining in these regions [44]. This expression pattern mirrors that of bone markers such as *col1a1a*, *col1a1b* and *col1a2*, which are involved in the formation of collagen chains crucial for bone development. These markers are expressed in zebrafish in similar developmental stages and in the same structures [45] where we observed the spatiotemporal expression of *ankha* and *ankhb*.

The expression of *ankha* and *ankhb* in the brain is particularly noteworthy as it implies potential roles beyond bone development. This is supported by human atlas proteome data, where ANK expression is prominent in oligodendrocytes and inhibitory neurons, tissues with high ATP demands [42]. Furthermore, mutations in *ANKH* in humans are associated with intellectual disability in CMD patients [46]. This raises the possibility that *ankha* and *ankhb* in zebrafish are also involved in neurological functions, emphasizing the need for a careful consideration of specific spatiotemporal roles when modeling CMD. These findings suggest that effective CMD modeling in zebrafish will require specific time- and tissue-specific gene knock-out and knock-in mutations [43] to accurately reflect the complexity of *ankha* and *ankhb* expression and to consider potential non-bone-related effects, particularly involving the brain.

The Gene Ontology (GO) functional analysis revealed that both Ankha and Ankhb proteins in zebrafish are predicted to have phosphate ion transmembrane transport and inorganic diphosphate transmembrane transport activities, functions that are also associated with other ANK proteins. This functional similarity underscores the potential of using zebrafish as a model to study the roles of these genes in bone mineralization processes and other developmental stages. The observed functional similarities among paralogs are not unique to *ankha* and *ankhb*. In zebrafish, *brd2* paralogs (*brd2a* and *brd2b*) play key roles in establishing egg polarity, transition from egg to embryo, development of the nervous system and formation of the digestive tract [40]. Similarly, *IGF* gene paralogs (*igf-1a*, *igf-1b*, *igf-2a* and *igf-2b*) exhibit functional redundancy, with mutations causing similar developmental issues in each gene knock-out [47]. However, zebrafish paralogs may not be entirely functionally redundant. In humans, mutations in the *SCN1A* gene causes severe childhood epilepsy, but there are two paralog genes in zebrafish, *scn1laa* and *scn1lab*. Disrupting one paralog mimics the heterozygous disease state, despite the other paralog still being present. While this ‘disease-state model’ is widely accepted, there is evidence suggesting that paralogous genes may not have identical functions [48].

## 5. Conclusions

Having determined that zebrafish Ankhb is more closely related to ANK/Ank in humans and mice, it is essential to validate these GO annotations to confirm whether Ankha and Ankhb are functionally similar, different or compensatory. However, due to the lack of specific antibodies for zebrafish Ankha and Ankhb, the functional roles of these paralogs remain speculative. Therefore, the most effective way to validate their functional roles is through the CRISPR-Cas9 system and the use of a more precise spatiotemporal control of CRISPR, later at the time of bone mineralization, to phenocopy CMD. Towards that end and given the similarity of Ankhb to human ANK, we hypothesize that *ankhb* knock-out mutants will exhibit more severe phenotypes compared to *ankha* mutants. Together, these findings highlight the necessity for further research using CRISPR-Cas9 to induce knock-out and knock-in mutations in *ankha* and *ankhb* corresponding to those found in human CMD. By generating these mutants, we will be able to study the resultant phenotypes to better understand the functional roles of and the potential compensatory mechanisms between these paralogs. These approaches will enhance our understanding of these *ankh* genes in CMD and in other events when bone is absent and support the development of targeted therapeutic interventions.

## Figures and Tables

**Table 1:** Primer sequences for zebrafish RT-PCR target genes.

Gene	Forward Primer (5′-3′)	Reverse Primer (5′-3′)
*ankha*	AGAAGCTTGCTTTCCTGTACCTCGCACT	TCGAATTCCAACCACCCATAGGGCATCT
*ankhb*	TAGAATTCCTTACATGGGGGTTCACGGG	AAGCTTGGAGCTGAGTTTTCGCCTTG
*lsm12b*	AGTTGTCCCAAGCCTATGCAATCAG	AGTTGTCCCAAGCCTATGCAATCAG

**Table 2:** Primer sequences for zebrafish RT-qPCR target genes.

Gene	Forward Primer (5′-3′)	Reverse Primer (5′-3′)
*ankha*	ATATAGCCATCGACTTCGGG	CTTGCCAGCATTTCAACCTT
*ankhb*	ACACATTTCCGAGAGCATCC	CGCGAATTGTCACTGGAATAG
*gapdh*	GTGGAGTCTACTGGTGTCTTC	GTGCAGGAGGCATTGCTTACA

**Table 3:** Primer sequences used for probe generation in zebrafish whole-mount in situ hybridization.

Gene	Forward Primer (5′-3′)	Reverse Primer (5′-3′)
*ankha*	AGAAGCTTGCTTTCCTGTACCTCGCAGT	TCGAATTCCAACCACCCATAGGGCATGT
*ankhb*	TAGAATTCCTTACATGGGGGTTCACGGG	ACAAGCTTGGAGCTGAGTTTTCGCCTTG

**Table 4:** Promoter prediction analysis results.

Promoter Predictions for Zebrafish *ankha*			
Start	End	Score	Promoter Sequence
29	79	0.84	AAATGTTTTATAAAAAAATTCCTAAAAAGTCACTAAAATTTTGTATTTTA
115	165	0.91	ACTTTTTGAGAAAAAAAGACCCACACCTTTTAACAGTCTGGCTCTGACCC
228	278	0.87	ATAAATGGCTTATAACAGTACCCCCAGACATTGTATTATATTGATTTATG
347	397	0.81	TGTCAATTTCTTTAAATAGCCTCCAGTAGAGGGCAGCATATATTTAAAAA
**Promoter Predictions for Zebrafish *ankhb***			
**Start**	**End**	**Score**	**Promoter Sequence**
215	265	0.96	CATGTGTTTATATAAAAGAAGTTTATGTGATTTATAAACCTTCAGTAACA
**Promoter Predictions for Human *ANKH***			
**Start**	**End**	**Score**	**Promoter Sequence**
228	278	0.82	GCCAGGGCACCCCGGGGTCTCCAGGCGGCCCACCGCCCTCACCCCCCACC
374	424	0.82	GAGGCGCCAGCCCCACGGCCCGAGCGTGCGCAGCGCCCCCCGCGGCCGCG
386	436	0.84	CCACGGCCCGAGCGTGCGCAGCGCCCCCCGCGGCCGCGCCAAGCGCAGGC
433	483	0.86	GGCGACGGCACAGGAAAGGAGGCCGCGGCGCGCCCGGCCCGGCCCCCTCC
461	511	0.87	CGCGCCCGGCCCGGCCCCCTCCCCAGCCCGCCCCCGGGGCCGCTGGCGGT
**Promoter Predictions for Mouse *Ank***			
**Start**	**End**	**Score**	**Promoter Sequence**
430	480	0.83	GCCCCCCGCGGCCGAGCCGTGCGCAGCGGAGCGGGGAGGCGGCGCCGGGC
515	565	0.83	CCGCCCGCCCCTGATTTCCTCCGCGCGGCGCGGCGGCGGCGGCGGAGGCG

**Table 5:** GO annotation results.

Protein	Biological Processes	Molecular Function	Cellular Component
ANK (*Homo sapiens*)	Phosphate ion transmembrane transport (GO:0035435)	Inorganic phosphate transmembrane transporter activity (GO:0005315)	Membrane (GO:0016020)
Ank (*Mus Musculus*)	Phosphate ion transmembrane transport (GO:0035435)	Inorganic phosphate transmembrane transporter activity (0005315)	Membrane (GO:0016020)
ankha (*Danio rerio*)	Phosphate ion transmembrane transport (GO:0035435)	Inorganic phosphate transmembrane transporter activity (GO:0005315)	Membrane (GO:0016020)
ankhb (*Danio rerio*)	Phosphate ion transmembrane transport (GO:0035435)	Inorganic phosphate transmembrane transporter activity (GO:0005315)	Membrane (GO:0016020)

## Data Availability

Data is included in this MS.

## Supplementary Materials

The following supporting information can be downloaded at: https://www.mdpi.com/article/10.3390/jdb12030023/s1. Figure S1: Multiple sequence alignment of the amino acid sequences of human (*ANKH*), mouse (*Ank*), and zebrafish (*ankha* and *ankhb*).
