# Supplementary material for: Evolution and Spatiotemporal Expression of ankha and ankhb in Zebrafish
Source: J Dev Biol. 2024 Sep 9;12(3):23. doi: 10.3390/jdb12030023 (PMC11417794; doi:10.3390/jdb12030023)
Supplement: Supplementary file 1 [file jdb-12-00023-s001.zip › Supplementary Figure 1 for Submission.docx]

Supplementary Figure 1


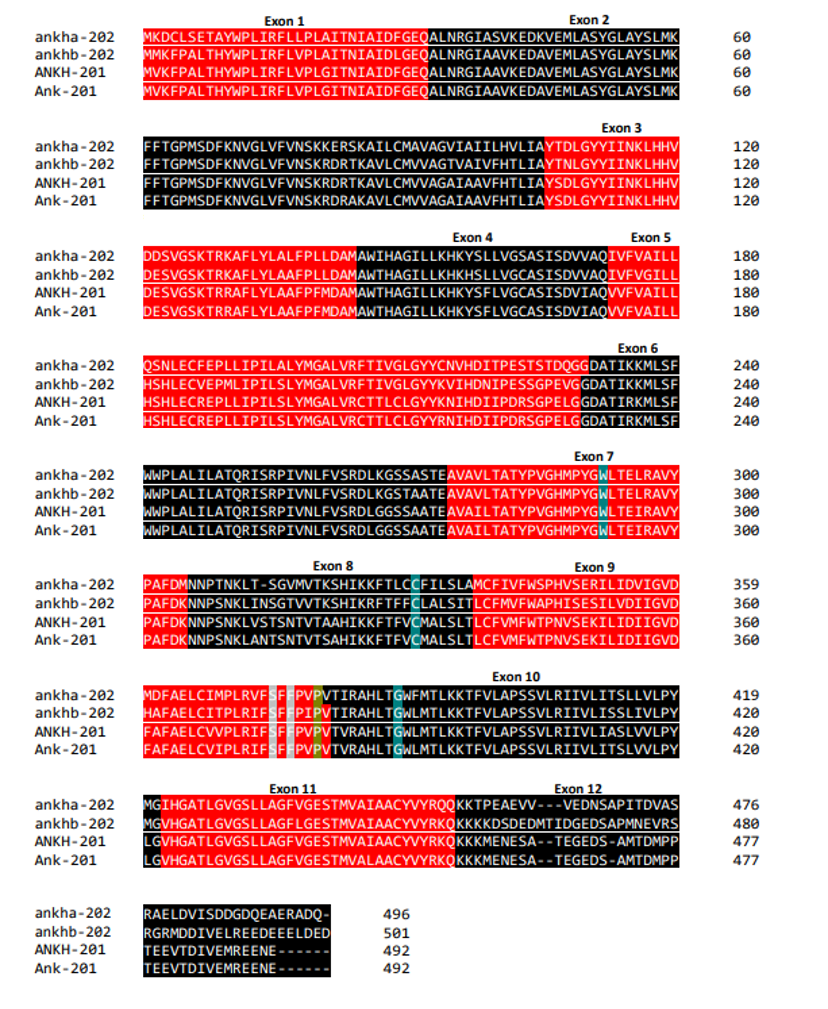


v

v

**Figure 1:** Multiple sequence alignment of the amino acid sequences of human (ANKH), mouse (Ank), and zebrafish (ankha and ankhb). Clustal Omega was used for multiple alignments of amino acid sequences obtained from the Ensmbl 112, 2023 database. For clear identification, exons are represented alternatively in red and black. The most common amino acid residues that cause mutations found in CMD patients [6] are highlighted in gray (deletions), yellow (insertions), and green (missense mutations).
